# A Hyperthermostable Archaeal GH78 Rhamnosidase Efficiently Hydrolyzes Flavonoid Glycosides for Juice Debittering

**DOI:** 10.1021/acs.jafc.5c16422

**Published:** 2026-02-10

**Authors:** Ali Shaikh-Ibrahim, Federica De Lise, Nicola Curci, Marika Gargano, Oriana Sacco, Mauro Di Fenza, Marco Moracci, Beatrice Cobucci-Ponzano

**Affiliations:** † Institute of Biosciences and BioResources, 9327 National Research Council of Italy, via P. Castellino, 111, Naples 80131, Italy; ‡ Department of Biology, 9307 University of Naples Federico II, via V.C. Cintia 26, Naples 80126, Italy; § Task Force on Microbiome Studies, University of Naples “Federico II”, Naples 80138, Italy; ∥ NBFC, National Biodiversity Future Center, Palermo 90133, Italy

**Keywords:** α-L-rhamnosidase, naringin, citrus juice, flavonoids, archaea, thermostable enzymes, industrial biocatalysis, carbohydrate-active enZymes

## Abstract

α-L-Rhamnosidases are a class of glycosyl hydrolases (GHs) that catalyze the hydrolysis of terminal α-L-rhamnose residues from diverse glycoconjugates. While extensively characterized in bacterial and fungal sources, no archaeal α-L-rhamnosidases have been characterized to date. Herein, we report the identification and characterization of the first thermostable archaeal α-L-rhamnosidase (ArRha), derived from the metagenomic data set of Pisciarelli solfatara hot spring. ArRha, classified in glycoside hydrolase family GH78, efficiently hydrolyzes α-1,2 and α-1,6 rhamnosyl linkages in flavonoid glycosides with notable biological activities. The novel enzyme showed remarkable temperature stability, wide-range pH activity, organic solvent tolerance, and no metal dependence. Combined with a thermostable β-glucosidase, ArRha converts naringin to prunin and naringenin in sweet and blood orange juices, achieving >95% conversion within 2 h at 65 °C. This represents the first report of a hyperthermostable archaeal GH78 α-L-rhamnosidase with promising applications in industrial enzymatic juice debittering and sustainable flavonoid biotransformation.

## Graphical abstract

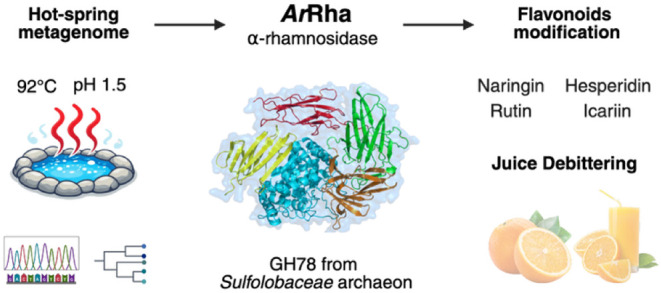

## 1 Introduction

Enzymes serve as nature’s precision catalysts, driving efficient and selective biotransformation that can be used in sustainable industrial processes. Their ability to operate under mild conditions, minimize waste, and generate high-value products from pharmaceuticals to food ingredients, positions enzymatic conversion as a cornerstone of green chemistry.^1^ Within this landscape, α-L-rhamnosidases hold strong potential as biocatalysts, particularly in valorizing flavonoid compounds. α-L-rhamnosidases are ubiquitous in nature and are found in animals, plants, and microorganisms. Notably, despite advances in bacterial and fungal α-L-rhamnosidase identification, characterization, and exploitation archaeal α-L-rhamnosidases are unknown to date. Based on amino acid sequence similarity, α-L-rhamnosidases of microbial origin are classified mainly into families GH78 and GH106 in the CAZy database.^2^ These enzymes hydrolyze the nonreducing end of α-L-rhamnose of rhamnose-containing polysaccharides and glycoconjugates found in various plant and microbial sources. They are categorized based on the linkage they cleave in α-1,2-, α-1,3-, α-1,4-, and α-1,6-rhamnosidases. Glycoconjugates substrates for α-L-rhamnosidases include flavonoid glycosides such as naringin, rutin, quercitrin, hesperidin, icarin, as well as nonflavonoid glycosides such as terphenyl glycosides.^3^ The specific cleavage of rhamnosides from natural flavonoids, results in compounds with enhanced bioavailability and bioactivity, feature that well meet biotechnological interests.^3,4^ Notable examples include prunin, hesperetin 7-O-glucoside, and isoquercitrin, which are valuable compounds in several sectors ranging from food, nutraceutical, cosmeceutical, and pharmaceutical.^3,5–7^ One of the biotechnological applications in the food sector is related to the use of α-L-rhamnosidases to reduce the bitterness of orange juices caused by the presence of naringin, which often exceeds the sensory threshold (≥30 mg/L).^6,8,9^ This application is particularly relevant because orange juice is one of the most widely consumed beverages worldwide, representing a significant portion of the global fruit juice market.^10,11^ Before the advent of enzymatic treatments, various techniques have been explored, including adsorption and ultrafiltration; however, these methods often result in nutrient loss or incomplete removal of bitterness.^12,13^ Instead, α-L-rhamnosidases have proven to be one of the most effective debittering methods due to their specific hydrolysis of naringin into prunin, which is subsequently hydrolyzed by a β-d-glucosidase to yield naringenin. Both prunin and naringenin are significantly less bitter compounds than naringin.^14,15^ In addition to naringin, other flavonoid glycosides such as neohesperidin, which also contribute to bitterness in certain citrus varieties (e.g., bitter orange and Seville orange), can be effectively transformed by α-L-rhamnosidases. For example, enzymatic debittering with *Aspergillus niger* α-L-rhamnosidase converts neohesperidin to less bitter hesperetin glucosides.^4^ Beyond improving the sensory properties of citrus juices, this enzymatic treatment can also enhance their nutritional value by increasing antioxidant levels and improving flavonoid bioavailability.^16^ Fungal α-L-rhamnosidases are suitable for biotechnological processes requiring high catalytic efficiency under acidic conditions, such as citrus juice debittering and clarification. These enzymes often possess some of the desirable traits, such as stability at elevated temperatures or tolerance to organic solvents; however, finding a single enzyme that combines all of the optimal characteristics, including broad pH tolerance, high thermal stability, and activity in harsh industrial conditions, remains challenging. For instance, the α-L-rhamnosidase from *Aspergillus tubingensis* ^17^ and *Spirochaeta thermophila* ^18^ exhibited optimal activity at 55 and 65 °C, respectively, close to the optimal temperatures of thermostable α-L-rhamnosidases from *Lactobacillus plantarum* WCFS1 (70 °C),^19^ *Aspergillus terreus* CCF3059 (65 °C), *and* *Alternaria alternata* SK37.001 (60 °C).^20,21^ However, only the enzyme from *S. thermophila* remains stable at temperatures above 65 °C, while it has a narrow pH range for activity. Although α-L-rhamnosidases from *Thermotoga* sp. exhibit the highest optimum temperatures and thermostabilities reported to date, their dependence on metal ions makes them not optimal for citrus debittering applications.^22^ These observations underscore the need to identify alternative enzymes with suitable stability, broad pH range activity, organic solvent tolerance, and metal independence. In this context, enzymes from Archaea could offer an additional advantage, as they are often naturally adapted to extreme conditions.^23–26^ Therefore, the search for new enzymes combining these properties remains a significant research goal. In recent years, metagenomic studies in extreme environments have led to the discovery of a wide array of CAZymes,^27^ highlighting their potential to be exploited in biotechnological applications. These enzymes, often exhibiting high stability, activity under extreme conditions, or novel substrate specificities, represent a valuable resource for developing new biocatalysts and industrial processes.^24–26,28,29^ The metagenome of the Solfatara Pisciarelli site (Agnano, Naples, Italy), an extremely acidic and high-temperature environment, provides access to unexplored archaeal CAZymes. Among the predicted sequences, a putative GH78 α-L-rhamnosidase was identified, representing the first archaeal enzyme of this class to be described. This study aims to characterize this previously unknown GH78 member and addresses the current lack of experimentally validated rhamnosidases from extremophiles while establishing its relevance for biotechnological applications in the agri-food sector.

## 2 Materials and Methods

### 2.1 Materials

All commercially available substrates were purchased from Sigma-Aldrich and Biosynth, unless otherwise stated. The pET-28a (+) plasmid containing the gene of interest was purchased from Twist Bioscience (California, USA).

### 2.2 Sequence Analysis

The sequences of GH78 and GH106 were obtained from the CAZy database (https://www.cazy.org/) and GenBank. Multiple sequence alignments were performed using Clustal Omega (v1.2.4) with default parameters, and alignment outputs were rendered with ESPript.^30^ Phylogenetic trees were visualized using iTOL (Interactive Tree Of life).^31^ The three-dimensional structure of ArRha was predicted through AlphaFold3.^32^ Visualization and analysis of the structural model were performed using the PyMOL Molecular Graphics System, Version 3.0, Schrödinger, LLC.^33^

### 2.3 Cloning, Expression, and Purification of ArRha

The incomplete gene encoding GH78 was first identified in the Pool2 metagenomic data set of the Solfatara Pisciarelli hot spring, Naples, Italy.^27^ However, the complete ORF sequence was obtained from an online metagenomic data set with the GenBank number (MCY0860088.1) and cloned into a pET-28a (+) vector purchased from Twist Bioscience. The resulting vector, pET-28a (+) contains *arrha*, with a 6x­(His) tag at the *N*-terminal region and kanamycin resistance. *E. coli* Lemo21­(DE3) competent cells transformed with pET-28a (+) *arrha* were grown in 2 L of terrific broth (tryptone 12 g/L, yeast extract 24 g/L, glycerol 0.4% v/v, potassium dihydrogen phosphate 0.017, and 0.072 M potassium phosphate dibasic) at 37 °C, supplemented with kanamycin (50 μg/mL) and chloramphenicol (20 μg/mL). Gene expression was induced by adding 0.5 mM IPTG when the culture reached an OD_600 nm_ of 0.5–0.6. Then, the growth continued for 16 h, and cells were harvested by centrifugation at 3,500*g* for 25 min at 4 °C. The resulting cell pellet was resuspended in 50 mM sodium phosphate buffer, pH 8.0, 300 mM NaCl, and Triton 1% (v/v) in a ratio of 1:5 (w/v). It was incubated with lysozyme (0.2 mg/mL) for 1 h, then 30 min with DNase (20 μg/mL) at room temperature, and then homogenized by a Multi Cycle (MC) Cell Disruptor (Constant Systems, UK) at 20 KPSI. The free cell extract was collected by centrifugation (25 min, 12,500*g* and 4 °C) and loaded onto an ÄKTA Explorer FPLC system (GE Healthcare) equipped with a HisTrap FF crude column (1 mL) (Cytiva, USA), previously equilibrated with 10 column volumes (CV) with Buffer A (50 mM sodium phosphate buffer, pH 8.0, 300 mM NaCl). After an initial wash step of 10-CV with buffer A, the elution was performed with a discontinuous gradient of buffer A supplemented with 500 mM imidazole (buffer B) as follows: 10 CV at 10% B, 10 CV at 50% B, and 10 CV at 100% B. The resulting fractions containing the enzyme were concentrated and loaded onto a HiLoad 16/600 Superdex 200 pg gel filtration column (GE Healthcare) that had been previously equilibrated with PBS buffer (20 mM Na_3_PO_4_, 100 mM NaCl, pH 7.3) at a flow rate of 1 mL/min (1.5 CV). The active fractions were pooled and stored at 4 °C. Protein concentration was determined by the Bradford method with bovine serum albumin (BSA) as a standard.^34^

### 2.4 Molecular Mass Determination

The molecular mass of ArRha was determined by size exclusion chromatography (SEC) using a Superdex 200 10/300 column (Cytiva); the enzyme storage buffer was used as the mobile phase with a flow rate of 0.5 mL/min. The molecular weight markers used were thyroglobulin (669 kDa), ferritin (440 kDa), aldolase (158 kDa), conalbumin (75 kDa), ovalbumin (44 kDa), carbonic anhydrase (29 kDa), ribonuclease A (13.7 kDa), and blue dextran (2000 kDa). A 200 μL aliquot of 0.75 mg/mL ArRha was loaded into the column. The native molecular mass was determined using a log_10_ molecular weight versus elution volume calibration curve generated from standard proteins.

### 2.5 GH78 Activity Assays

To determine the substrate specificity, ArRha was screened on several 4NP-substrates (α-L-Rha, α-L-Araf, α-L-Arap, α-L-Fuc, β-L-Fuc, β-D-Glc, β-D-Gal, α-D-Man, and α-D-Xyl). The standard assay of ArRha was performed in 50 mM sodium acetate buffer, pH 6.5, at 75 °C using 5 mM 4NP-α-L-Rha in a total volume of 200 μL, with 1 μg of purified enzyme. A blank mixture without the enzyme was used as a control. The reaction mixture was preincubated at 75 °C for 2 min, after which the enzyme was added and incubated for 3 min. The reaction was stopped by directly adding 800 μL of 1 M Na_2_CO_3_ (pH 10.2) and transferred to ice. The absorbance was spectrophotometrically detected at 420 nm at room temperature. The mM extinction coefficient of 4NP under these condition is 17.2 M^–1^ cm^–1^. One unit of enzyme activity was defined as the amount of enzyme releasing 1 μmol of 4NP under standard reaction conditions.

### 2.6 The Effect of pH and Temperature on ArRha Activity

The temperature dependence of the activity of ArRha was determined in the range of 45–100 °C in 50 mM sodium acetate buffer (pH 6.5) with 4NP-α-L-Rha (5 mM) as the substrate in the standard assay conditions. Optimal pH was determined by assaying GH78 in the standard assay conditions using 50 mM sodium acetate buffer (pH 3.5–6.5), 50 mM sodium phosphate buffer (pH 6.5–9.5), and 50 mM glycine-NaOH buffer (pH 9.5–10.5). The thermal stability was evaluated by incubating the enzyme in PBS buffer (pH 7.3) at 55, 65, 75, and 85 °C for up to 48 h. Aliquots (5 μg) were withdrawn at the indicated times and assayed in triplicate under the standard conditions described above. The residual activity was expressed as a percentage of the maximal enzymatic activity measured before incubation with values representing the mean of replicates. Structural thermal stability was assessed by differential scanning fluorimetry (DSF) according to Iacono et al., 2025 ^35^ with slight modifications. The enzyme (0.075 mg/mL) was incubated in storage buffer (PBS, pH 7.3) with 5x SYPRO Orange in a final volume of 50 μL using a Step One Plus Real-Time PCR System. Samples were incubated at 25 °C for 10 min and subjected to a temperature ramp from 25 to 95 °C at 0.1 and 1 °C min^–1^. Protein unfolding was monitored by SYPRO Orange fluorescence, which was normalized to the maximum signal of each scan. Each value represents the mean of two independent experiments performed at least in triplicate. Melting temperature (*T*_m_) was calculated according to Niesen et al., 2007.^36^

### 2.7 Kinetic Parameters

The kinetic parameters of the enzyme were determined on 4NP-α-L-Rha in a range of 0.05–20 mM in standard conditions, as described above. Additionally, under the same assay conditions but with a 20-min incubation, kinetic analyses were performed on 0.05–6 mM naringin, 0.1–10 mM rutin, 0.025–6 mM hesperidin, and icariin at concentrations ranging from 0.5 to 4 mM. All assays have been blocked directly in ice, and the reaction mixtures were filtered through 0.22 μm membranes before analysis. Enzymatic activity on flavonoids was quantified by measuring the release of L-rhamnose using high-performance anion-exchange chromatography with pulsed amperometric detection (HPAEC-PAD). Analyses were performed on a Dionex CarboPac PA1 column with 20 mM NaOH as the mobile phase at a flow rate of 0.75 mL/min, and fucose (2.5 nmol) served as an internal standard.

### 2.8 Effect of Chemicals and Organic Solvents on Catalytic Activity

To determine the effect of metal ions on ArRha activity, 1 mM and 10 mM final concentrations of the following metals (K^+^, Zn^2+^, Ca^2+^, Mg^2+^, Na^+^, Cu^2+^, and Fe^3+^) and EDTA were added to the reaction mixture. The relative activity was calculated as a percentage compared to that of the enzyme under standard assay conditions. In addition, the effect of organic solvents methanol, ethanol, and DMSO, with final concentrations of 5%, 10%, and 20% (v/v) was also evaluated, assaying enzymatic activity in standard conditions.

### 2.9 Naringin Biotransformation

Naringin hydrolysis was tested at 65 and 75 °C in sodium acetate buffer (pH 6.5), for 1 and 2 h, in a final volume of 0.5 mL. The reaction mixture contained 2 mM of naringin (∼1.2 mg/mL) and 2.5 μg of either ArRha or LacS, a thermophilic GH1 enzyme from *Saccharolobus solfataricus*,^37^ or in combination. Each condition was tested with and without the enzymes as a control, and all assays were performed away from light. After incubation, each reaction was stopped on ice and centrifuged at 12,000 g, 4 °C for 5 min. The supernatant was filtered using a 0.22 μm membrane, and the release of rhamnose and glucose was determined using an HPAEC-PAD system (Dionex ICS-6000). Analyses were performed on a Dionex CarboPac PA1 column (4 × 250 mm) using isocratic elution with 20 mM NaOH at a flow rate of 1 mL/min for 15 min at 35 °C. Fucose (2.5 nmol) served as an internal standard. In addition, the reaction was analyzed for the detection of prunin and naringenin using a high-performance liquid chromatography (HPLC) system (LC-4000 Series System by Jasco, Oklahoma, OK, USA), with a Teknoroma Mediterranea Sea 18 column (25 × 0.46 mm, 5 μm) and a UV/vis detector (Jasco UV 4070 detector) was used to monitor the absorbance at 280 nm. The mobile phase was composed of water with 0.1% trifluoroacetic acid (TFA) (phase A) and acetonitrile with 0.1% TFA (phase B). The elution was carried out at a flow rate of 1.0 mL/min using the following gradient: 0–4 min, A/B = 90:10; 4–23 min, A/B = 30:70; 23–24 min, A/B = 30:70; and 24–30 min, A/B = 90:10. The quantification of naringin, prunin, and naringenin was performed by running several concentrations of pure substrate as standards (calibration curve: naringin (5–150 μM); prunin (0.5–150 μM), naringenin (0.5–50 μM). All samples were filtered through a 0.22 μm filter and diluted 1:20 in 10% B before injection.

### 2.10 Orange Juice Debittering

The debittering potential of ArRha was evaluated using two orange varieties: sweet orange (*Citrus sinensis*, SWO) and blood orange (*Citrus sinensis* L. Osbeck, BLO). The fresh juice from each variety was collected immediately after extraction without industrial processing. The pH was measured, and samples were stored at −20 °C, protected from light to maintain quality before enzymatic treatment. Small-volume assays were conducted by mixing juice and reaction mixtures at sample-to-reaction ratios of 1:5, 2:5, and 3:5 (v/v) to a final volume of 0.5 mL. Each mixture (pH 6.5) was incubated at 65 °C for 2 h with either 2.5 μg of ArRha alone or combined with 2.5 μg of LacS (β-glucosidase), using a thermomixer at 500 rpm with protection from light. Reactions were stopped by rapidly cooling the sample on ice. Larger-volume assays were performed at a 25 mL final volume (3:5 ratio) at 65 °C for 2 h, using 5 μg/mL of each enzyme. After incubation, samples were filtered through 0.22 μm membranes and centrifuged at 12,000*g* for 5 min to remove particulates. Resulting supernatants were appropriately diluted and analyzed by HPLC for naringin, prunin, and naringenin identification and quantification following the previously reported protocol. Additionally, the debittering potential of ArRha was tested without buffering the reaction mixtures.

### 2.11 Statistical Analysis

All enzyme activity assays, temperature and pH profiles, substrate specificity, metal dependence, and solvent tolerance experiments were conducted with three independent replicates. Naringin biotransformation and juice debittering experiments were performed in duplicate. Data are presented as mean ± standard deviation (SD). Kinetic parameters were determined from duplicate measurements and calculated by nonlinear regression analysis using the Michaelis–Menten equation in GraphPad Prism software (version 10.0, San Diego, CA, USA). Statistical significance of differences between experimental conditions for metal ion dependence and solvents was assessed using two-way analysis of variance (ANOVA), followed by Sidak’s and Dunnett’s multiple comparison tests, respectively. Differences were considered statistically significant at *p* < 0.05. All data processing, graphical representations, and statistical analyses were performed using GraphPad Prism software.^38^

## 3 Results and Discussion

### 3.1 Sequence Analysis and Alpha-Fold Prediction

An incomplete sequence encoding a putative α-L-rhamnosidase was identified in the metagenomic data set of Pool 2 from Solfatara Pisciarelli, Agnano (Naples, Italy).^27^ The full-length sequence was retrieved from a metagenomic data set of the same hydrothermal site (BioSample: SAMN31719908) and designated as *arRha*. The gene encoding putative GH78 (GenBank accession No. MCY0860088.1), annotated as an uncharacterized α-L-rhamnosidase from a *Sulfolobaceae* archaeon, comprises a 2592 bp full-length gene encoding an 863-amino acid protein (predicted MW: 100.2 kDa; pI: 5.75). To date, the GH78 family includes 15,031 putative rhamnosidase sequences from bacterial and eukaryotic sources, of which only 30 are of archaeal origin; however, the *arRha* gene is not among them. To date, only 49 GH78 rhamnosidases have been functionally characterized, none of which are from archaea. To investigate the evolutionary relationship between ArRha and the α-L-rhamnosidases of the GH78 family, a phylogenetic tree was constructed using the neighbor-joining method based on multiple sequence alignments incorporating functionally characterized GH78 and GH106 α-L-rhamnosidases as reference sequences (Figure 1A). The ArRha enzyme was clustered closely with two enzymes from thermophilic bacteria, *Thermotoga petrophila* RKU-1 ^39^ and *Dictyoglomus thermophilum* H-6–12.^40^ Both enzymes showed activity on 4NP-α-L-Rha and natural substrates, such as naringin and rutin. GH78 members follow a catalytic *inverting* mechanism, as demonstrated for the thermostable α-L-rhamnosidase RamA from *Clostridium stercorarium*.^41^ This catalytic mechanism was further confirmed by the inspection of the crystal structures of *Streptomyces avermitilis* and *D. thermophilum* (PDB ID: 3W5M and 6I60, respectively).^40,42^ Despite the relatively low degree of sequence identity (∼42%) with other characterized GH78 members, ArRha retains two conserved catalytic motifs: IPTDCPQRDERMGW (residues 438–451) and TTLWERWE (residues 713–720) (Figure S1), which include the conserved putative catalytic residues Glu^447^ and Glu^717^. The AlphaFold3 predicted model of ArRha (Figure 1B), obtained by using AlphaFold3,^32^ shows the presence of five distinct domains: one α-domain (domain A) and four β-domains, labeled N, E, F, and C, as shown in Figure 1C. Domain A functions as the catalytic module, adopting an (α/α)_6_-barrel configuration commonly found in GH78 family enzymes, while the surrounding N, E, F, and C domains comprise primarily β-strands. ArRha conserves the catalytic motif located within domain A, including the characteristic arrangement of two glutamic acid residues (Glu^447^ and Glu^717^) involved in acid/base catalysis, with strong structural homology to the well-characterized *Aspergillus terreus* GH78 α-L-rhamnosidase (RMSD: 1.8 Å; Figure S2).^43,44^ The structural superposition between ArRha and the *A. terreus* rhamnosidase highlights conserved catalytic regions and similar folding patterns, supporting a comparable catalytic role (Figure S2).

**Figure 1:**
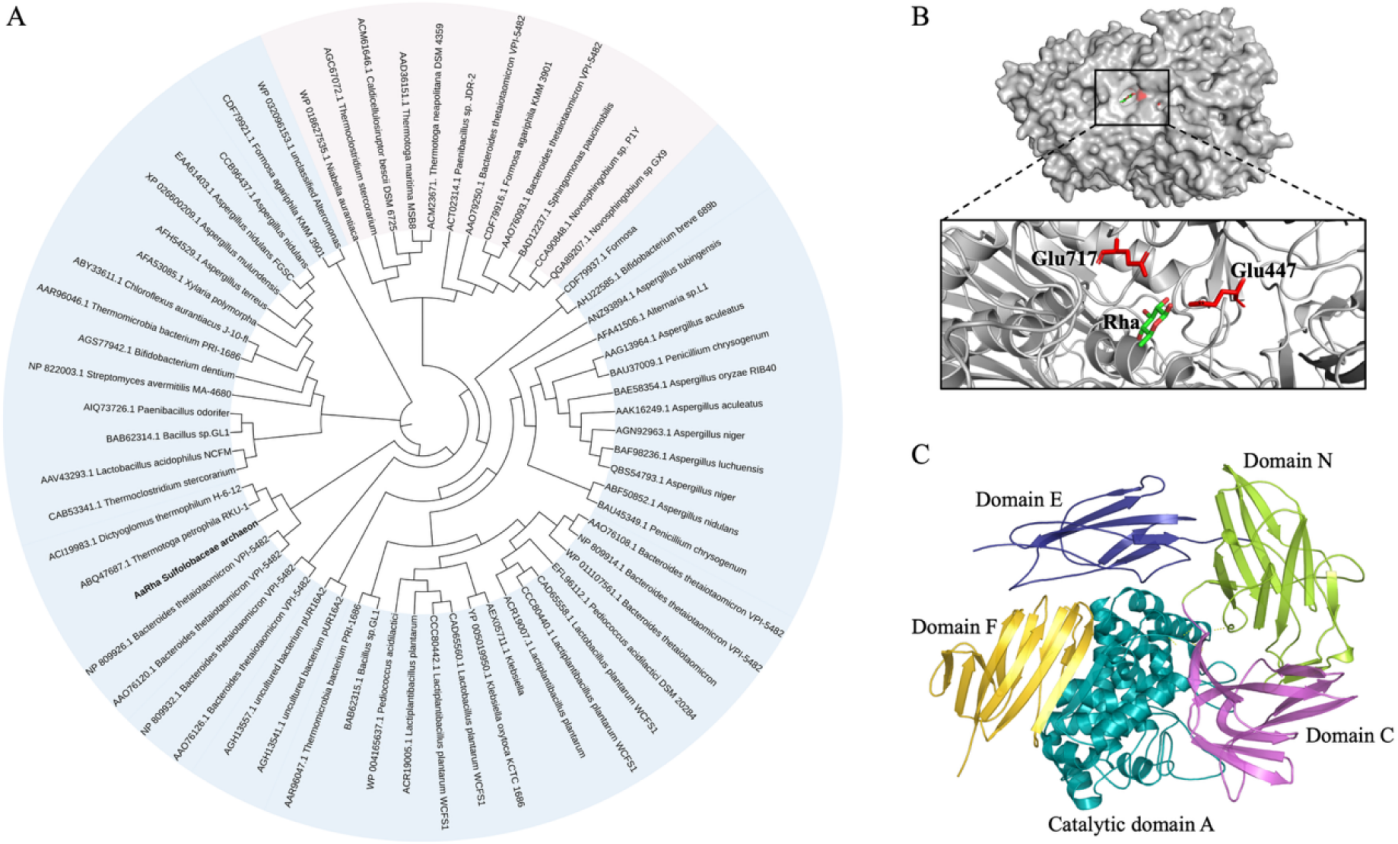
(A) Phylogenetic tree of GH78 (light blue area) and GH106 (light pink area) α-L-rhamnosidases. ArRha is highlighted in bold. Gene bank accession numbers and microorganism names represent individual α-L-rhamnosidases. (B) The overall predicted 3D structure of ArRha is depicted, with the putative catalytic acid/base residues Glu^447^ and Glu^717^ highlighted in red. (C) The structure is organized into distinct domains, colored and named according to the PFAM database annotation.

The accessory domains’ composition varies among α-L-rhamnosidases, with only domain F consistently shared among all homologues with known three-dimensional structures. Even though the precise functions of the accessory domains (N, E, and C) remain unclear, their structural variability among GH78 enzymes suggests they may contribute to enzyme stability, substrate positioning, or molecular recognition, rather than being directly involved in catalysis.^40,44^ Indeed, several bacterial and fungal α-L-rhamnosidases lacking these domains still retain full catalytic activity, such as the two-domain α-L-rhamnosidase KoRha from *Klebsiella oxytoca*,^45^ supporting the idea that they are not essential for enzymatic turnover.

### 3.2 Expression and Purification of the Recombinant Enzyme

The *ArRha* gene was cloned into the plasmid pET-28a (+) in frame with the sequence encoding a six His-tag at the 5'-end. The gene was expressed in *E. coli* and the resulting enzyme was purified through a two-step chromatography process, as described in the Materials and Methods section. The analysis of the denaturing gel confirmed the purity of the protein, as it migrates as a single band at approximately 96 kDa, compatible with the predicted molecular mass of the monomer (100.2 kDa) (Figure S3). The production yield of the protein is in the range of 0.9–1 mg/L culture (Table S1). The molecular mass of ArRha under native conditions is 368 ± 3 kDa, indicating a homotetrameric quaternary structure (Figure S4). The majority of GH78 enzymes are typically monomeric or dimeric, and few characterized enzymes adopt higher-order oligomeric states, such as α-L-rhamnosidases from *Lactobacillus plantarum* ^19^ and *RhaA* of *Bacillus* sp. strain GL1.^46^ It is worth mentioning that ArRha remains stable when stored at 4 °C in sodium phosphate buffer (pH 7.3, 100 mM NaCl), with full retention of enzymatic activity over six months.

### 3.3 Biochemical Characterization

Among all the tested 4NP-derived substrates, namely, α-L-Rha, α-L-Araf, α-L-Arap, α-L-Fuc, β-L-Fuc, β-D-Glc, β-D-Gal, α-D-Man,and α-D-Xyl, ArRha showed activity only on 4NP-α-Rha. The enzymatic activity of ArRha increased up to 100 °C, the highest tested, reaching up to 32 ± 2 U/mg (Figure 2A).

**Figure 2:**
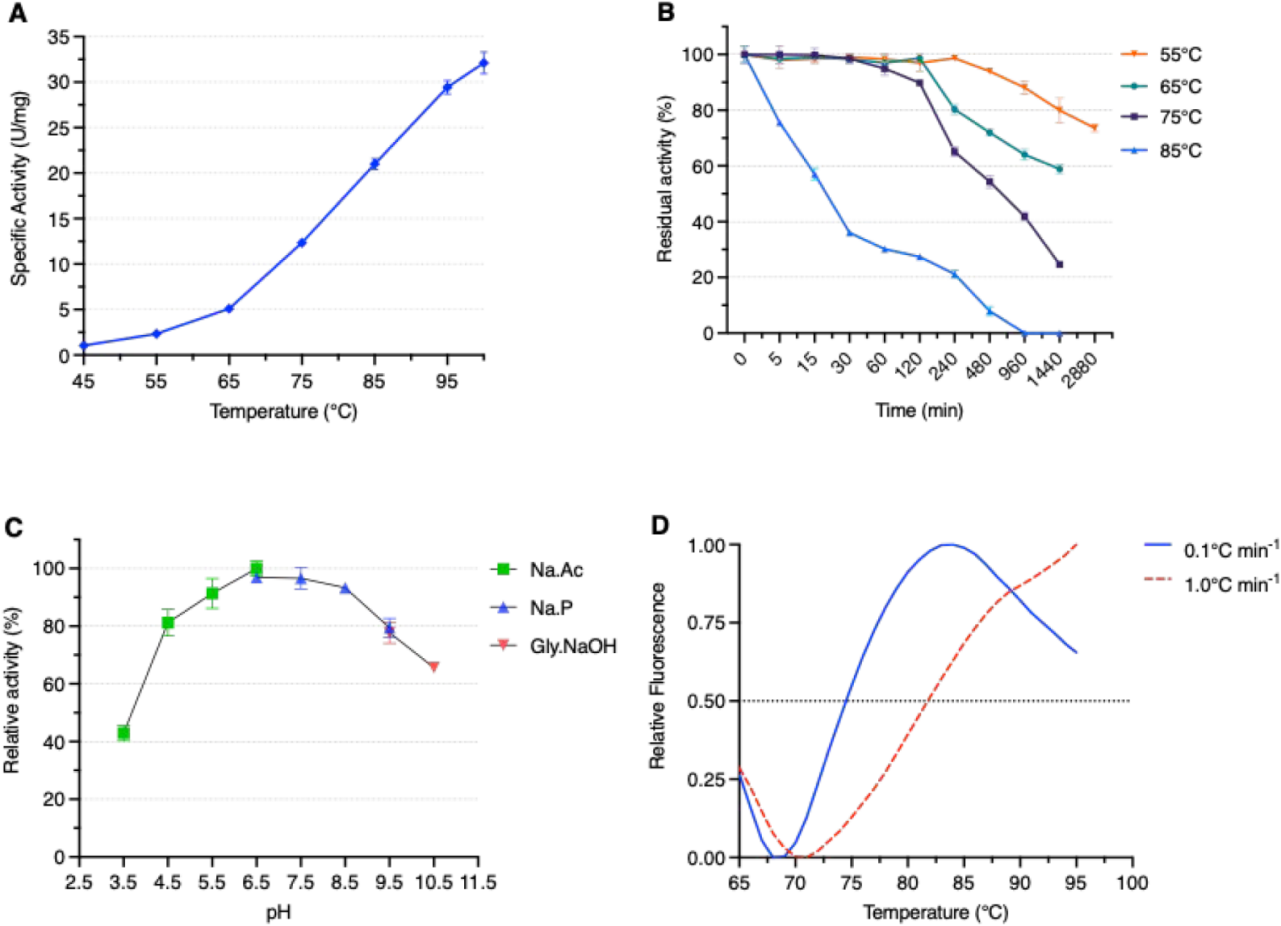
Effect of (A) temperature on ArRha activity, (B) temperature on ArRha stability, and (C) pH on ArRha activity. The initial activity was defined as 100%, and subsequent measurements of enzyme activities were expressed as relative values. All the assays were performed on 4NP-α-L-Rha. (D) Thermal stability profiles of ArRha determined by DSF.

Additionally, thermal stability measurements demonstrate that the enzyme maintained 100% residual activity for up to 4 h at 55 °C, 2 h at 65 °C, and 1 h at 75 °C (Figure 2B). This thermal profile is comparable to that of the α-L-rhamnosidase from *D. thermophilum* (DtRha) and *T. petrophila* (TpeRha), which also display markedly higher optimum temperatures than those reported for bacterial and fungal counterparts, reaching 90 and 95 °C, respectively.^39,40^ The optimum pH range for most bacterial α-L-rhamnosidases is 5.0–8.0, and it is generally in the range of 5.0–6.5 for fungal α-L-rhamnosidases.^3^ While the optimal pH for the enzymatic activity of ArRha was determined to be 6.5 (Figure 2C), the enzyme retained 80% of its maximum activity over a wide pH range from 4.5 to 9.5, broader than *Papiliotrema laurentii* rhamnosidase, which also showed a broad pH range (5.5–9.0).^47^ Thermal unfolding profiles showed that ArRha is a highly stable enzyme. The *T*_m_ of 75 °C was determined using a slow heating rate of 0.1 °C min^–1^, as faster scanning rates did not allow accurate determination, as shown in Figure 2D. The enzyme activity has been also tested on the natural substrates (hesperidin, naringin, rutin, and icariin), and the steady-state kinetic constants on these different substrates are reported in Table 1 and Figure S5. ArRha shows the highest affinity for naringin and the highest turnover number for 4NP-α-Rha, and achieves the highest catalytic efficiency on hesperidin, with a *k*_cat_/*K*_M_ value of 121.9 ± 3.1 s^–1^mM^–1^ . It is also worth noting that ArRha is able to hydrolyze icariin (α-1) at 0.5 to 4 mM, showing a specific activity of 0.2 U/mg at 1 mM; however, detailed kinetic parameters could not be determined as this substrate is only soluble in DMSO, which inhibits the enzyme at high concentrations.

**Table 1:** Substrate Specificity and Kinetic Parameters of ArRha on 4NPαLRha and Flavonoid rhamnosides^a^

**Substrate**	**Type of linkage**	***K***_**M**_ **(mM)**	***k***_**cat**_ **(s** ^**–1**^ **)**	***k***_**cat**_ **/** ***K***_**M**_ **(s** ^**–1**^ **mM** ^**–1**^ **)**
**Hesperidin**	α-1,6	0.26 ± 0.01	31.7 ± 0.6	121.9 ± 3.1
**4NP-α-Rha**	α-1	0.56 ± 0.02	54.1 ± 2.1	96.6 ± 7.1
**Naringin**	α-1,2	0.13 ± 0.04	10.7 ± 0.2	82.0 ± 3.6
**Rutin**	α-1,6	1.50 ± 0.12	31.9 ± 0.7	21.3 ± 1.5

a Values are mean ± SD. Assays were performed at 75 °C, pH 6.5, see Materials and Methods section.

The enzymological characterization allowed us to confirm that ArRha is indeed an α-L-rhamnosidase, the first characterized from Archaea, with clear activity on α-1, α-1,2, and α-1,6 linkages. The high catalytic efficiency, thermostability, and wide range of pH activity make ArRha particularly interesting for industrial applications. In particular, the high catalytic efficiency toward hesperidin and naringin underscores its potential for industrial applications in the biotransformation of flavonoid glycosides.^8,48^

### 3.4 Metals, EDTA, and Organic Solvents Effect

The effects of metal ions, EDTA, and organic solvents on recombinant ArRha activity were evaluated under standard conditions of the assay, by using 5 mM 4NP-α-L-Rha as the substrate (Figure 3A and B).

**Figure 3:**
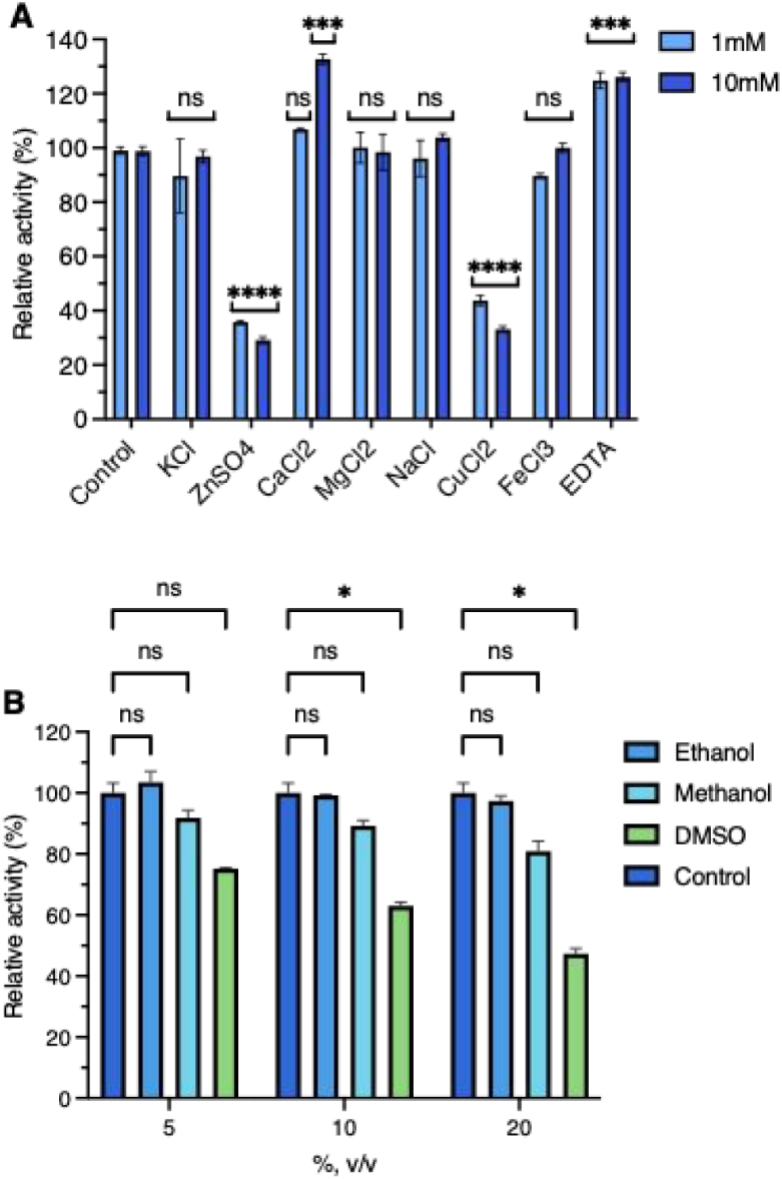
(A) The effect of metal ions and EDTA and (B) organic solvents on ArRha activity. Statistical significance was assessed by two-way ANOVA with Dunnett’s multiple comparisons vs control (buffer + enzyme) and Sidak’s for selected pairwise comparisons (ns: not significant, *P* > 0.05, *: *P* ≤ 0.05, **: *P* ≤ 0.01, ***: *P* ≤ 0.001, ****: *P* ≤ 0.0001).

Metal ions K^+^, Mg^2+^, Na^+^, and Fe^3+^ exhibited negligible or minimal effect on enzymatic activity at both 1 and 10 mM concentrations. Conversely, Cu^2+^ and Zn^2+^ strongly inhibited the activity at both concentrations, reducing it to ≤50%. Cu^2+^/Zn^2+^ inhibition aligns with that of *Bacillus* sp. GL1 rhamnosidase, where these ions disrupt catalytic residues via covalent bonding.^49^ Conversely, Ca^2+^ acted as a mild activator, enhancing activity to 130% at 10 mM concentration, consistent with observations in some bacterial and fungal α-L-rhamnosidases such as those from *Bacteroides thetaiotaomicronas* ^50^ and *Absidia sp.90*.^51^ The role of Ca^2+^ in the catalytic process was suggested by comparing the crystal structures of the five available α-L-rhamnosidases. When the substrate binds to the active site of the enzyme, Ca^2+^ can form coordination bonds with O3 and O4 of the substrate rhamnose, thereby facilitating the catalysis.^42,52^ The chelator EDTA induced a slight activity increase (∼20%) at 1 mM, with no additional effect at 10 mM, indicative of possible trace metal contaminants inhibiting ArRha, in accordance with what was reported for *D. thermophilum* DtRha.^40^ Overall, the data show that ArRha displays no dependence on metal ions, in contrast with what has been observed for GH106 α-L-rhamnosidases, which showed strong metal ion dependency.^22,53,54^ The tolerance of α-L-rhamnosidases to organic solvents (ethanol, methanol, and DMSO) is one of the key factors for their use as a natural and efficient catalyst in the bioconversion of flavonoids, since most of them are more soluble or only soluble in these solvents. ArRha shows remarkable tolerance to organic solvents, retaining 100% of its activity in the presence of up to 20% (v/v) ethanol and 80% in 20% (v/v) methanol. In the presence of 20% (v/v) DMSO, ArRha maintains approximately 50% activity (Figure 3B). It is worth noting that stability assays in 20% (v/v) of these solvents have also been conducted for one h, yielding the same results as activity assays, indicating that ArRha’s high solvent tolerance is sustained over time. This hierarchy of solvent resilience (ethanol > methanol > DMSO) underscores ArRha’s suitability for nonaqueous biocatalytic systems, particularly those requiring ethanol or methanol to solubilize hydrophobic substrates. ArRha’s solvent tolerance exceeds that of fungal counterparts such as *A. niger*, which retains about 50% activity in 15% methanol.^15^ The DMSO higher sensitivity of ArRha mirrors that of *T. petrophila*, where it has been suggested that hydrogen bond disruption destabilizes catalytic loops.^39^ Table 2 compares the key parameters of ArRha with GH78 α-L-rhamnosidases with optimum temperatures ≥55 °C and biochemical characterization, selected as representative thermostable enzymes.

**Table 2:** Enzymatic Properties and Kinetic Parameters of Characterized GH78 α-L-Rhamnosidases with Optimal Activity Temperatures ≥55 °C^a^

Enzyme/Organism	Temp Opt (°C)	pH > 50% activity	*K*_M_ (mM)	*k*_cat_(s^– 1^)	*k*_cat_/*K*_M_ (s^– 1^ mM^– 1^)	Thermostability	Organic solvent (methanol)	ref
ArRha/*S. archaeon*	100	4.0 – 10	0.56	54.1	97	>50% activity after incubation at 75 °C for 8 h	80% activity at 20% methanol	This study
DtRha/*D. thermophilum*	95	4.0–7.5	0.05	0.2	3.1	>50% activity after incubation at 70 °C for 60 min	57% activity at 20% methanol	^40,55^
TpeRha/*T. petrophila* *DSM*	90	4.5–5.0	3.00	651.0	220	>50% activity after incubation at 80 °C for 80 min	83% activity at 15% methanol	^39^
AoRhaA/*A. oryzae*	70	3.0–8.0	1.40	1.1	0.8	>50% activity after incubation at 60 °C for 60 min	NR*	^56^
RhmB/*T. bacterium* *PRI-1686*	70	4.0–7.9	0.66	NR*	1.9	>50% activity after incubation at 60 °C for 8 h	NR*	^57^
RhmA/*T. bacterium* *PRI-1686*	70	5.0–8.7	0.46	NR*	1	>50% activity after incubation at 60 °C for 4 h	NR*	^57^
Ram2/*P. acidilactici* *DSM 20284*	70	4.0–7.0	22.30	3.7	0.2	Loss of activity after incubation at 70 °C after 10 min	NR*	^58^
RhaL1/*Alternaria sp. L1*	70	4.5–9.5	1.36	NR*	NR*	95% activity after a 2 h incubation below 60 °C, not stable after 60	NR*	^59^
St-Rha*/* *S. thermophila*	65	5.5–6.5	12.70	5.6	0.4	>50% activity after incubation at 75 °C for 6 h	94% activity at 30% methanol	^18^
TstRhaA/*T. stercorarium* *subsp.thermolacticum DSM 2910*	65	5.5–7.5	0.36	650	1810	>60% activity after incubation at 60 °C for 4 h	50% activity at 20% methanol	^60^
AmRha/*A. mulundensis* *DSM 5745*	65	4.7–7.5	0.30	900	2969	>60% activity after incubation at 55 °C for 2 h	50% activity at less than 10% methanol	^61^
Rha1/*A. niger*	60	4.0–8.5	NR*	NR*	NR*	>50% activity after incubation at 60 °C for 5 h	85% activity at 20% methanol	^62^
RhaB2/*L. plantarum* *NCC245*	60	4.5–5.5	0.51	NR*	NR*	>50% activity after incubation at 55 °C for 2h	NR*	^63^
Rha78A/*B.thetaiotaomicron* *VPI-5482*	55	6.0–7.5	2.90	1743	607.4	>50% activity after incubation at 50 °C for 2 h	NR*	^50,53^
B689b_0522/*B. breve* *689b*	55	5.0–7.5	2.20	2.5	0.9	>90% activity after incubation at 60 °C for 20 h, loss stability	NR*	^64^
						>70 (half-life 25 min)		
AT-rRha/*A. tubingensis* *JMU-TS529*	55	3.0–6.5	0.47**	48.9**	102**	>50% activity after incubation at 65 °C for 30 min	NR*	^17^

a *NR: not reported. Kinetic parameters were determined using 4NP-α-Rha as the substrate under the standard conditions reported for each enzyme in the respective reference. ** Kinetic parameters were determined using naringin as substrate.

ArRha stands out among GH78 members by combining high thermostability, tolerance to organic solvents, metal independence, and the broadest pH range of activity. These characteristics indicate a robust enzyme that retains activity under challenging conditions and remains stable for at least six months at 4 °C, suggesting its suitability for long storage and the possibility of reuse in multiple cycles, which may enhance its practical applicability. As the first thermostable archaeal GH78 α-L-rhamnosidase to be biochemically characterized, ArRha provides valuable insight into this GH family and broadens the repertoire of candidates suitable for industrial bioprocesses.

### 3.5 Naringin Biotransformation

Building on the distinctive biochemical features of ArRha, its potential in biocatalytic applications was investigated through the biotransformation of flavonoids. The conversion of naringin represents a key step toward the synthesis of products used in the pharmaceutical, cosmetic, and food industries, as well as a crucial process for the development of enzymatic debittering strategies for citrus juices. To evaluate the catalytic ability of ArRha to hydrolyze naringin, its conversion to prunin was assessed. Subsequently, the transformation of prunin to naringenin was investigated by combining the activities of ArRha and LacS, a thermostable β-glucosidase from *Saccharolobus solfataricus* (Figure 4).^37,65^ LacS (β-glycosidase, SSO3019) has been previously characterized for its robust activity under extreme conditions and successfully employed in the deglycosylation of various glycoconjugates, including stevioside and other natural glycosides.^37,66^ Similar to the β-glucosidase from *Pyrococcus furiosus*, which has been used to produce flavanone aglycones from citrus extracts,^67^ the combination of ArRha and LacS enables complete enzymatic debittering by sequentially removing both rhamnose and glucose moieties from naringin, thereby producing the nonbitter aglycone naringenin.

**Figure 4:**
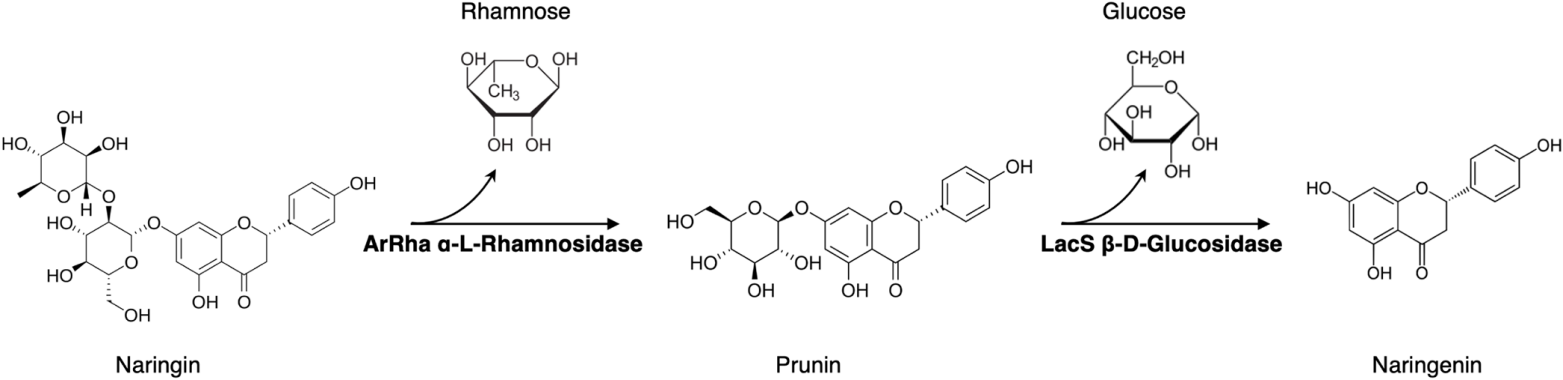
Schematic representation of the enzymatic biotransformation of naringin.

The reactions were carried out at 65 and 75 °C for 1 and 2 h. HPAEC-PAD analyses allowed for the detection and quantification of the released rhamnose and glucose after each reaction (Table 3 and Figure S6).

**Table 3:** Rhamnose and Glucose Were Obtained After the Action of ArRha and LacS on Naringin

	Enzyme	Time (min)	Rha nmol (mean ± SD)	Glu nmol (mean ± SD)
65 °C	ArRha	60	155.4 ± 1.0	ND^a^
	LacS	60	23.6 ± 0.6	24.4 ± 1.4
	ArRha and LacS	60	171.2 ± 0.3	128.2 ± 4.0
	ArRha	120	264.3 ± 0.4	ND^a^
	LacS	120	43.8 ± 1.4	48.8 ± 3.4
	ArRha and LacS	120	273.7 ± 0.4	216.5 ± 0.4
75 °C	ArRha	60	356.3 ± 23.9	ND^a^
	LacS	60	65.9 ± 2.0	124.7 ± 10.9
	ArRha and LacS	60	366.2 ± 7.1	278.4 ± 4.2
	ArRha	120	550.6 ± 2.5	ND^a^
	LacS	120	123.5 ± 2.1	142.8 ± 0.8
	ArRha and LacS	120	584.8 ± 2.5	456.4 ± 1.1

a *ND: not detected.

As reported in Table 3, ArRha alone hydrolyzed naringin, releasing rhamnose. The combined action of ArRha and LacS, at 75 °C for 2 h, resulted in the release of approximately 584.8 nmol of rhamnose (67% yield) and 456.4 nmol of glucose (65% yield), relative to the maximum amounts of sugars released by complete sulfuric acid hydrolysis of the same amount of naringin (877.7 ± 30.3 nmol rhamnose and 700.4 ± 5.9 nmol glucose), demonstrating that both enzymes are highly efficient in naringin conversion at elevated temperatures. Remarkably, LacS was found to release rhamnose, providing the first evidence that this enzyme can hydrolyze α-L-rhamnosides, although much less efficiently than ArRha. To further confirm and quantify the reaction products, the same reactions were analyzed by HPLC to determine the concentrations of naringin, prunin, and naringenin (Table 4).

**Table 4:** Naringin, Prunin, and Naringenin Quantified by HPLC Analyses

	**Enzyme**	**Time (min)**	**Naringin nMol (mean ± SD)**	**Prunin nMol (mean ± SD)**	**Naringenin nMol (mean ± SD)**
	Blank reaction		1085 ± 93	ND^a^	ND
**65 °C**	ArRha	60	732 ± 94	156 ± 16	ND
	LacS	60	1022 ± 19	ND	ND
	ArRha and LacS	60	700 ± 48	ND	55 ± 0.89
	ArRha	120	470 ± 40	293 ± 13	ND
	LacS	120	880 ± 14	28 ± 3	19 ± 4
	ArRha and LacS	120	450 ± 30	76 ± 7	116 ± 4
**75 °C**	ArRha	60	482 ± 70	211 ± 25	ND
	LacS	60	900 ± 30	ND	25 ± 2
	ArRha and LacS	60	389 ± 13	ND	65 ± 8
	ArRha	120	237 ± 32	378 ± 24	ND
	LacS	120	825 ± 52	ND	39 ± 5
	ArRha and LacS	120	255 ± 35	ND	164 ± 12

a *ND: not detected. Percentages (w/w) are calculated considering the initial weight of naringin.

As reported in Table 4, after 2 h at 75 °C, ArRha converted 74% of naringin, yielding 38% prunin. Interestingly, the combined action of ArRha and LacS converted 75% of naringin into naringenin, with a yield of 16%, indicating that prunin produced by ArRha was completely transformed into naringenin by LacS under these conditions. Notably, the measured prunin and naringenin yields underestimate the actual amount present due to precipitation and filtration losses during sample preparation, as supported by HPLC analysis and previous reports on flavonoid recovery limitations.^68^ In addition, note that no attempts at further optimization were performed. These results confirm the high catalytic efficiency of both enzymes at elevated temperatures and demonstrate their effective cooperation in completing the enzymatic conversion of naringin. Furthermore, this cooperation, and the possibility of using the two enzymes either in combination or back-to-back, could be advantageous for tailoring the efficient hydrolysis of naringin toward the selective production of prunin or naringenin instead of using on direction process such as in the naringinase from *A. aculeatus*.^69^ The activity of ArRha on several flavonoids highlights its potential as a biocatalyst for tailored biotransformations aimed at improving the solubility, bioavailability, and sensory properties of flavonoid in various biotechnological applications.

### 3.6 Orange Juice Debittering Efficiency

The bitter taste of citrus juices is primarily due to the presence of flavonoid glycosides such as naringin, hesperidin, and neohesperidin. Enzymatic debittering of citrus juices primarily targets the bitter flavonoid naringin, using naringinases, dual-activity enzyme complexes that combine α-L-rhamnosidase and β-d-glucosidase activities to hydrolyze naringin into the nonbitter compounds prunin and naringenin. It is well known that α-L-rhamnosidase activity alone or in combination with β-d-glucosidase can increase the floral flavor of orange juice, and the combined treatment has a more higher effect on the taste and aroma quality of juice.^70–72^ This process typically achieves 33–36% bitterness reduction under optimized conditions (4 h at 40–50 °C), with enzyme immobilization being a key factor in further enhancing efficiency.^73^ Furthermore, prunin and naringenin retain higher antioxidant activity than naringin, enhancing juice nutraceutical value without bitterness.^74,75^ To evaluate the debittering potential of ArRha and LacS, enzymatic treatments were applied to fresh blood orange (BLO, pH 3.8) and sweet orange (SWO, pH 3.6) juices under controlled conditions, and the naringin, prunin, and naringenin were quantified. Initial naringin concentrations were approximately 0.69 mg/mL in BLO and 0.87 mg/mL in SWO (Table 5).

**Table 5:** HPLC Analysis of Small-Scale Juice Debittering Trials Assays Using ArRha and LacS^a^

Blood orange *Citrus sinensis* L. Osbeck (BLO)		
Sample	Naringin (mg/mL)	Prunin (mg/mL)
BLO	0.69 ± 0.09	ND
Blank	0.76 ± 0.05	ND
ArRha	0.12 ± 0.02	0.30 ± 0.01
ArRha and LacS	0.11 ± 0.01	ND

Sweet orange *Citrus sinensis* (SWO)		
Sample	Naringin (mg/mL)	Prunin (mg/mL)
SWO	0.87 ± 0.08	ND*
Blank	0.68 ± 0.09	ND
ArRha	0.07 ± 0.03	0.28 ± 0.05
ArRha and LacS	ND	ND

a *ND: not detected, meaning no measurable amounts of compound.

After 2 h of incubation at pH 6.5 and 65 °C, HPLC quantification indicated 85% of naringin conversion in BLO by using an enzyme loading of 5 mg/L for each enzyme. Interestingly, a nearly complete conversion of naringin was obtained in SWO under the same conditions. The results reported in Table 5 were obtained by using a juice-to-reaction mixture ratio of 3:5; similar conversion yields were obtained with 1:5 and 2:5 juice-to-reaction mixture ratios. The combined action of ArRha and LacS showed high debittering efficiency in comparison to previously reported systems. For instance, the immobilized *Penicillium decumbens* naringinase showed >90% conversion in 8 h at 50 °C with an enzyme loading of 500 mg/L juice.^76^ Similarly, free naringinase from *A. niger* required 60 min at 40 °C to achieve approximately 90% naringin reduction in honey pomelo juice with 100 U enzyme per 100 mL juice.^72^ ArRha achieved complete naringin conversion in 2 h at 65 °C with a lower enzyme loading (5 mg/L) (15 U), positioning it among the most efficient biocatalysts for juice processing. Furthermore, the elevated temperature activity and stability offer additional advantages for industrial applications, including reduced microbial contamination risk and enhanced reaction kinetics. To further evaluate the performance of ArRha and LacS under more application-relevant conditions, a scale-up experiment was carried out by increasing the reaction volume 50-fold, maintaining the same conditions described above. This approach aimed to assess the enzyme’s catalytic efficiencies and to determine the conversion yield at a larger, laboratory scale.

The results in Figure 5 clearly show that ArRha nearly achieved complete conversion of naringin, as revealed by the disappearance of the relative peak, into prunin. Furthermore, the combined action of ArRha and LacS converted the naringin into naringenin. These results were obtained with both juices (Figure 5A,B), clearly indicating the ability of the two enzymes to function in higher lab-scale conditions. Additionally, the ability of ArRha to catalyze naringin hydrolysis directly in juice at low pH values (3.6 and 3.8) was evaluated, revealing that the enzyme is able to convert naringin under these conditions, achieving a conversion yield of approximately 40% (data not shown).

**Figure 5:**
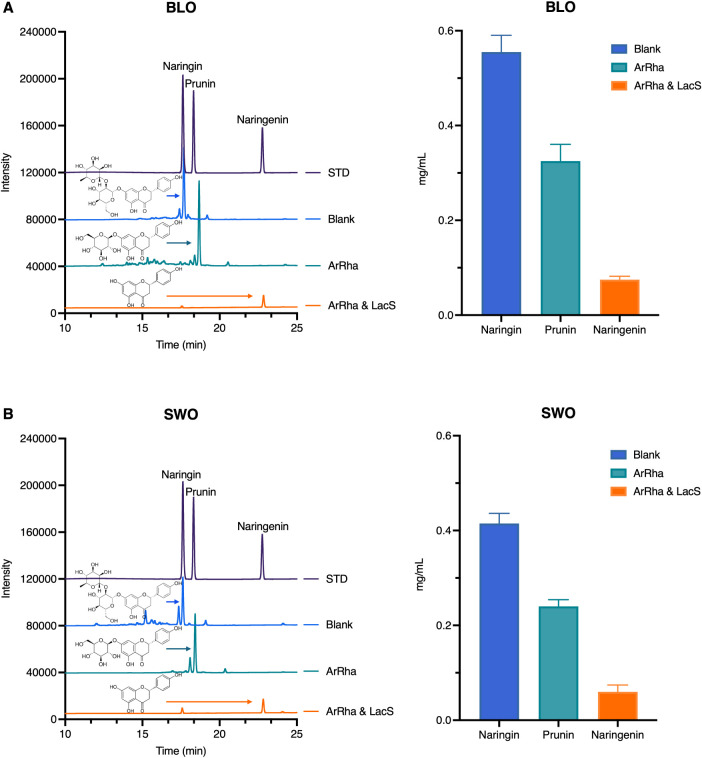
HPLC chromatograms show the identification of naringin, prunin, and naringenin and their quantification (A) for BLO and (B) for SWO.

This work highlights the remarkable potential of enzymes derived from metagenomes of extreme environments as novel biocatalysts for biotechnology applications. Despite the growing number of CAZyme sequences identified through metagenomic studies, only a small fraction has been functionally characterized, particularly those from Archaea. Given the harsh environmental conditions in which (hyper)­thermophilic Archaea thrive, archaeal enzymes have evolved to function under extreme conditions of temperature, pH, and salinity, making them especially attractive for industrial processes that require robustness and stability. Their thermostability and resistance to denaturation reduce the need for strict process controls to increase reaction efficiency, while their tolerance to acidic or otherwise harsh conditions opens the way for innovative bioprocesses. Future research directions may focus on expanding the discovery and characterization of enzymes from extreme environments, alongside mutagenesis studies and the design of multienzymatic synergistic systems to enhance the efficiency of transformation processes. Building on this concept, ArRha represents the first biochemically characterized archaeal GH78 α-L-rhamnosidase, providing valuable insights into the diversity of archaeal CAZymes and expanding our understanding of their catalytic potential. The distinctive properties of this enzyme also demonstrate its suitability for applications such as enzymatic debittering in the citrus juice industry and, more broadly, for sustainable bioprocess development and innovative biotechnological applications yet to be explored.

## Supplementary Material



## Notes

Multiple sequence alignment analysis, FPLC chromatogram, purification yield table, supporting figures of kinetics, and HPAEC-PAD chromatogram (PDF)

## Abbreviations

4NP-α-L-Araf: 4-Nitrophenyl-α-L-arabinofuranoside
4NP-α-L-Arap: 4-Nitrophenyl α-L-arabinopyranoside
SWO: Sweet orange *Citrus sinensis*
BLO: Blood orange *Citrus sinensis* *L.* Osbeck
Rha: Rhamnose
Glu: Glucose
4NP-α-Rha: 4-Nitrophenyl-α-L-rhamnopyranoside
4NP-β-Fuc: 4-Nitrophenyl-β-D-fucopyranoside
4NP-β-Gal: 4-Nitrophenyl-β-D-galactopyranoside
4NP-β-Glc: 4-Nitrophenyl-β-d-glucopyranoside
4NP-β-Man: 4-Nitrophenyl-β-D-mannopyranoside
4NP-β-Xyl: 4-Nitrophenyl-β-D-xylopyranoside
4NP: 4-nitrophenol
CAZymes: Carbohydrate-active enZymes
CV: column volumes
GH: glycoside hydrolase
IPTG: isopropyl-β-D-1-thiogalactopyranoside
LacS: a thermostable β-glycosidase enzyme from *Saccharolobus solfataricus*
ArRha: α-L-rhamnosidase of this study
TB: Terrific broth
