# Supplementary material for: A Hyperthermostable Archaeal GH78 Rhamnosidase Efficiently Hydrolyzes Flavonoid Glycosides for Juice Debittering
Source: J Agric Food Chem. 2026 Feb 10;74(6):5562–74. doi: 10.1021/acs.jafc.5c16422 (PMC12921842; doi:10.1021/acs.jafc.5c16422)
Supplement: Supplementary file 1 [file jf5c16422_si_001.pdf]

Supporting Information

A hyperthermostable archaeal GH78 rhamnosidase efficiently hydrolyzes flavonoid glycosides for juice debittering

Ali Shaikh-Ibrahim [a], Federica De Lise [a], Nicola Curci [a], Marika Gargano [b], Oriana Sacco [a,b], Mauro Di Fenza [a], Marco Moracci [b,c,d], Beatrice Cobucci-Ponzano [a*]

Affiliations

[a] *Institute of Biosciences and BioResources, National Research Council of Italy, via P. Castellino, 111, 80131 Naples, Italy*
[b] *Department of Biology, University of Naples Federico II, Via V.C. Cintia, 26, 80126 Naples, Italy*
[c] *Task Force on Microbiome Studies, University of Naples "Federico II" 80138, Naples, Italy* [d] *NBFC, National Biodiversity Future Center, 90133, Palermo, Italy*

*Corresponding author beatrice.cobucciponzano@cnr.it

```
WP_018627535.1_Niabella_aurantiaca                      378  .......ESSPNAQFGNIRSVKELSSVANQL..........GKERTL..CETYGGAGW...DLTFKDMKR
AGC67072.1_Thermoclostridium_stercorarium               365  .................YSTVKQAQSVARQF..........GKAGVL..SELYGVTNW...DFDFRGHKL
ACM61646.1_Caldicellulosiruptor_bescii_DSM_6725         314  .......Q.........VVTMKQVSSVAEQL..........GKKGVL..CETFGTTGQ...HVSFLHRKW
AAD36151.1_Thermotoga_maritima_MSB8                     313  .......Q.........VVTIKQVSSAAEQL..........GKKWVL..CETFGTTGQ...HVSFLHRKW
ACM23671._Thermotoga_neapolitana_DSM_4359               313  .......Q.........VITIKQVSSVAEQL..........RKKWVL..CETFGTTGQ...HVSFLHRKW
ACT02314.1_Paenibacillus_sp._JDR-2                      389  ....RNTSGEADSEFFHYGLAKLGVSLAHID..........PKKQGRTMCEVYGAYGW...TEGLKLMKW
AAO79250.1_Bacteroides_thetaiotaomicron_VPI-5482        364  ....DSLTKKNDS...DLSMLKYASSAAHIA..........GKPYTSTETFTWLTEHF...RTSLSQCKP
CDF79916.1_Formosa_agariphila_KMM_3901                  410  .......F.........FDSPKFVSSIAHLN..........GSSIVGAESFTG.IGGW...DEHPAELKS
AAO76093.1_Bacteroides_thetaiotaomicron_VPI-5482        567  .........PTHD...KPNDMLDAISGAHIY..........GKNIIQAEGFTEVRGTW...NEHPGILKA
BAD12237.1_Sphingomonas_paucimobilis                    592  .......SAPRPT...LIGDMKGAASVAHIY..........GQNIVSAESMTAAFSPW...AFAPADLKR
CCA90848.1_Novosphingobium_sp._P1Y                      616  .......EGPRQT...LIADMKGAASVAHLY..........GQNLVAAESMTASMAPW...AFAPKDLKR
QGA89207.1_Novosphingobium_sp_GX9                       628  .......TGPNPS...YLADMKGAASVAHVY..........GQNLVAAESMTSALAPW...AYTPKDLRR
AAO76126.1_Bacteroides_thetaiotaomicron_VPI-5482        505  .........................SVFTDCPHR.EKLGWLEQVHLNGPGLLYNYDLT...AYAPQIMQN
NP_809932.1_Bacteroides_thetaiotaomicron_VPI-5482       505  .........................SVFTDCPHR.EKLGWLEQVHLNGPGLLYNYDLT...AYAPQIMQN
AAO76120.1_Bacteroides_thetaiotaomicron_VPI-5482        488  .........................GMPVDCPQRNERQPWLGDHAMGSWGESMFFDNH...AMYNKWARD
NP_809926.1_Bacteroides_thetaiotaomicron_VPI-5482       488  .........................GMPVDCPQRNERQPWLGDHAMGSWGESMFFDNH...AMYNKWARD
AaRha_Sulfolobaceae_archaeon                            437  .........................GIPTDCPQRDERMGWLGDSNLTIEVAMFNLWAY...GFYREWIRQ
ABQ47687.1_Thermotoga_petrophila_RKU-1                  452  .........................GIPTDCPQRDERMGWLGDAQLTVEEAMYNFDMA...AFYTKYLMD
ACI19983.1_Dictyoglomus_thermophilum_H-6-12             469  .........................SIPTDCPQRDERMGWMGDAQLSAEEAIFNFDMI...GFYRKYLND
CAB53341.1_Thermoclostridium_stercorarium               436  .........................DVPTDCPQRDERMGWTGDAQVFSGAAAFNMDVF...AFFGKYLYD
AAV43293.1_Lactobacillus_acidophilus_NCFM               432  .........................DVPTDCPQRDERLGWTGDAEIFAPTASFNMNTY...EFYKKYAKD
CDF79921.1_Formosa_agariphila_KMM_3901                  477  .........................DIPTDCPQRDERMGWTGDAQVFGPTSMFNADVY...KFWASWMQS
WP_032096153.1_unclassified_Alteromonas                 496  .........................DIPLDCPQRDERLGWTGDANAFIAPSMYMADVY...GFWSAYLNS
NP_822003.1_Streptomyces_avermitilis_MA-4680            626  .........................SVPTDTPARDERLGWTGDINVFAPTAAYTMESA...RFLTKWLVD
BAB62314.1_Bacillus_sp.GL1                              459  .........................DVPTDCPQRDERLGWTGDAQAFVRASTYNRNVQ...SFFAKWLRD
AIQ73726.1_Paenibacillus_odorifer                       453  .........................DVPTDCPQRDERLGWTGDAQMFVRTASYLMNTA...PFFTKWLRD
AGS77942.1_Bifidobacterium_dentium                      480  .........................SVPQDCPQRDERMGWTGDICLFAPTAAYLYDVY...GFLKSWLKD
AAR96046.1_Thermomicrobia_bacterium_PRI-1686            453  .........................HIPTDCPQRDERLGWTGDIQVFSPAACFIYDAS...GFLTSWLRD
ABY33611.1_Chloroflexus_aurantiacus_J-10-fl             465  .........................DVPTDCPQRDERLGWTGDIQVFAPTAVFLYDVA...GLLQSWLID
AFA53085.1_Xylaria_polymorpha                           457  .........................SIPTDCPQRDERLGWTGDAHAFAPTANFLYNPA...GFWRGWMKD
AFH54529.1_Aspergillus_terreus                          457  .........................SIPTDCPQRDERLGWTGDIHAFSRTANFIYDTA...GFLRAWLKD
XP_026600209.1_Aspergillus_mulundensis                  454  .........................SIPTDCPQRDERLGWTGDINAFARTANFIYDTA...GFLRGWLKD
EAA61403.1_Aspergillus_nidulans_FGSC                    454  .........................SIPTDCPQRDERLGWTGDIHAFARTANFIYDTS...GFLRGWLRD
CCB96437.1_Aspergillus_nidulans                         454  .........................SIPTDCPQRDERLGWTGDIHAFARTANFIYDTS...GFLRGWLRD
CDF79937.1_Formosa                                      737  .........................NGLYVDGDR.ERIPYEADAYLNQLSHYTTDR...EYAMARRTIEY
AHJ22585.1_Bifidobacterium_breve_689b                   395  .........................PIYADSWTR.ERAPYEADAWLQQRSHLALDCGADAIRLGRYSVDW
ANZ93894.1_Aspergillus_tubingensis                      424  LY.RNP..MTLSSVLEEYGVMASKHSVCLDGAKR.DRLVWNGDFVHTYRVIQSSTYRS...DFITGSLEY
AFA41506.1_Alternaria_sp.L1                             223  GYSKDDDQTPVNVTWYNNYTITAGKSALVDGAKR.DRLVWAGDMAIAVPGVVVSTNDV...ISIENALDS
AAG13964.1_Aspergillus_aculeatus                        211  .S.SETISTSGLNYWYNNLTIANGTSTVTDGAKR.DRAVWPGDMSISLESIAVSTNDL...YSVRMGLEA
BAU37009.1_Penicillium_chrysogenum                      218  SS.GDNVHLPQTDTWWNNYTITNGSSTITDGAKR.DRLVWPGDMSIALETIAVSTGDL...YSVRTALES
BAE58354.1_Aspergillus_oryzae_RIB40                     221  SS.DSNITLPETNPWYSNYTISNGSSTLTDGAKR.DRLIWPGDMSIALESVSVSTADL...YSVRTALET
AAK16249.1_Aspergillus_aculeatus                        219  SS.SDNITLPQTDSWWNNYTITNGSTTLVDGAKR.DRLVWPGDMSIAIESAAVSTADL...ESVRTALES
AGN92963.1_Aspergillus_niger                            216  TS.SETITLPQTDKWWTNYTITNGSSTLTDGAKR.DRLVWPGDMSIALESVAVSTEDL...YSVRTALES
BAF98236.1_Aspergillus_luchuensis                       216  SS.SETISLPQTDKWWTNYTITNGSSTLTDGAKR.DRLVWPGDMSIALESVAVSTEDL...YSVRTALES
QBS54793.1_Aspergillus_niger                            216  TS.SETISLPQTDKWWTNYTITNGSSTLTDGAKR.DRLVWPGDMSIALESVAVSTEDL...YSVRTALES
ABF50852.1_Aspergillus_nidulans                         229  .......SVSSATGWNNNAVCGPGETLLLDGAKR.DRWVWIGDMGVAVPSASVSTGDT...ESTKNALLA
BAU45349.1_Penicillium_chrysogenum                      232  .......TKGVTSGWVNNGTLGPGDTIIVDGAKR.DRAVWPGDMGIAVPSAFVSLGDL...ESVKSALQI
AGH13541.1_uncultured_bacterium_pUR16A2                 989  .........................DTFFDCPER.ERAQWWGDAVVLMGECFYTYSTS.VHALMSKAIHE
AGH13557.1_uncultured_bacterium_pUR16A2                 651  .........................DTFFDCPER.ERAQWWGDAVVLMGECFYTYSTS.VHALMSKAIHE
AAO76108.1_Bacteroides_thetaiotaomicron_VPI-5482        326  .........................EFFIDGIKR.DRWVWSGDAIQSYLMNYYLFFDS...ESVKRTIWL
NP_809914.1_Bacteroides_thetaiotaomicron_VPI-5482       326  .........................EFFIDGIKR.DRWVWSGDAIQSYLMNYYLFFDS...ESVKRTIWL
WP_011107561.1_Bacteroides_thetaiotaomicron             326  .........................EFFIDGIKR.DRWVWSGDAIQSYLMNYYLFFDS...ESVKRTIWL
EFL96112.1_Pediococcus_acidilactici_DSM_20284           273  .........................LFFIDGIKR.DRWIWGGDAYQDNFINQYSFFNE...EVDRRTILA
CAD65558.1_Lactobacillus_plantarum_WCFS1                275  .........................LFFIDGIKR.DRWIWAGDAYQANFINQYSFFNE...DIDKRTLLA
CCC80440.1_Lactiplantibacillus_plantarum_WCFS1          275  .........................LFFIDGIKR.DRWIWAGDAYQANFINQYSFFNE...DIDKRTLLA
ACR19007.1_Lactiplantibacillus_plantarum                273  .........................LFFIDGIKR.DRWIWAGDAYQANFINQYSFFNE...DIDKRTLLA
BAB62315.1_Bacillus_sp.GL1                              563  .........................DTFVDCPSY.EQVFWVGDSRNEALVNYYVFGET...EIVERCLNL
AAR96047.1_Thermomicrobia_bacterium_PRI-1686            566  .........................DTFVDCPAY.EQTFWVGDSRNEALTAYYLFGAE...ELVRRCLRL
AEX05711.1_Klebsiella                                   213  .........................EVFEDGPKR.DRRLWLGDLRLQALVNDVTFARH...DLVRRCLYL
YP_005019950.1_Klebsiella_oxytoca_KCTC_1686             213  .........................EVFEDGPKR.DRRLWLGDLRLQALVNDVTFARH...DLVRRCLYL
CAD65560.1_Lactobacillus_plantarum_WCFS1                217  .........................DVFEDGPKR.DRRLWIGDLRLQALANYATFKDT...DLVKRCLYL
CCC80442.1_Lactiplantibacillus_plantarum_WCFS1          214  .........................DVFEDGPKR.DRRLWIGDLRLQALANYATFKDT...DLVKRCLYL
ACR19005.1_Lactiplantibacillus_plantarum                214  .........................DVFEDGPKR.DRRLWIGDLRLQALANYATFKDT...DLVKRCLYL
WP_004165637.1_Pediococcus_acidilactici                 213  .........................DVFEDGPKR.DRRLWIGDLRLQALADYQTYDNQ...TLIKRCLYL
```

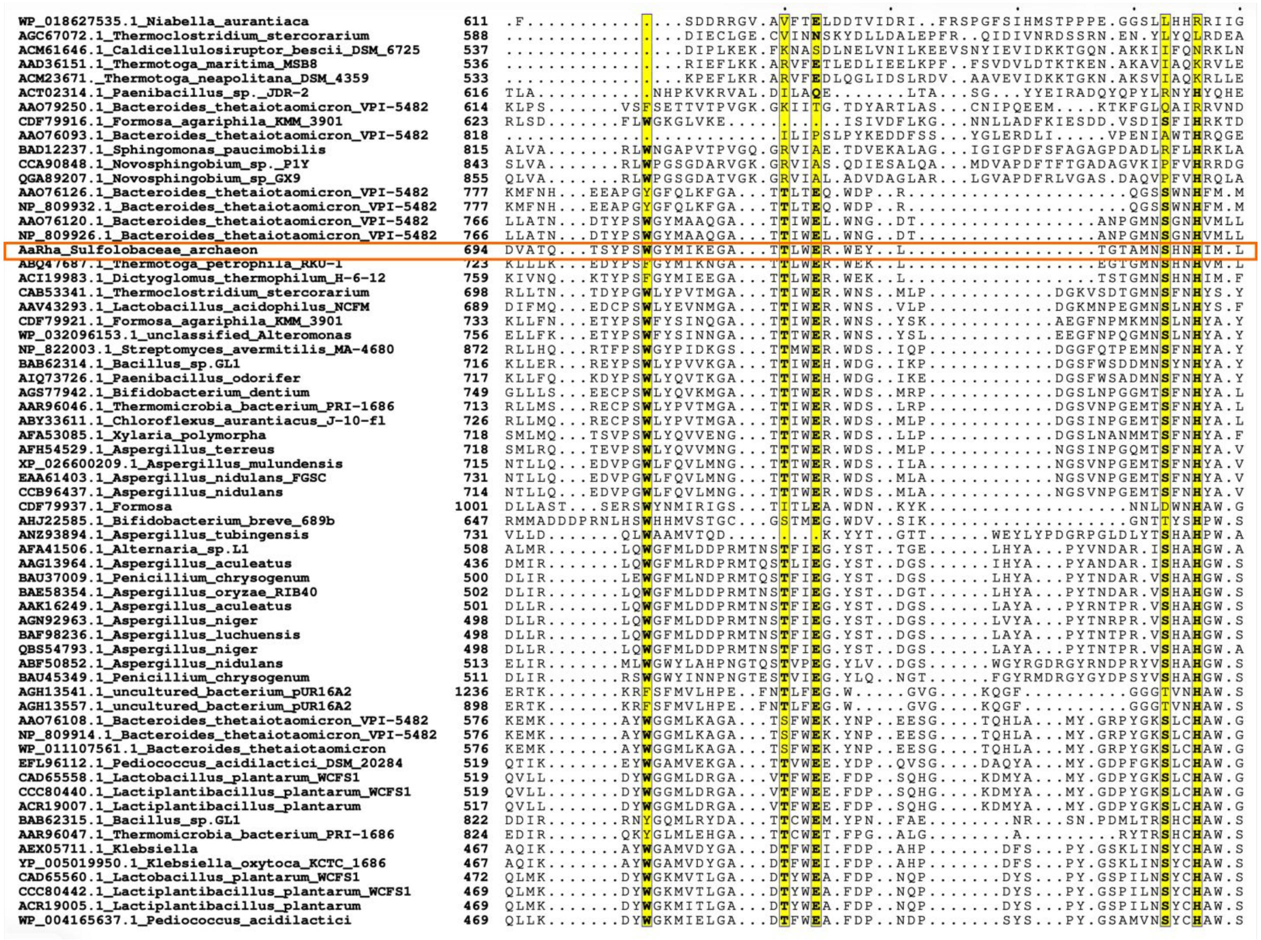

Figure S1. Comparison of the aminoacidic sequence of ArRha (orange box) with characterized GH78 α-L-rhamnosidases . Gene bank accession numbers and microorganism names represent the individual α-L-rhamnosidases.

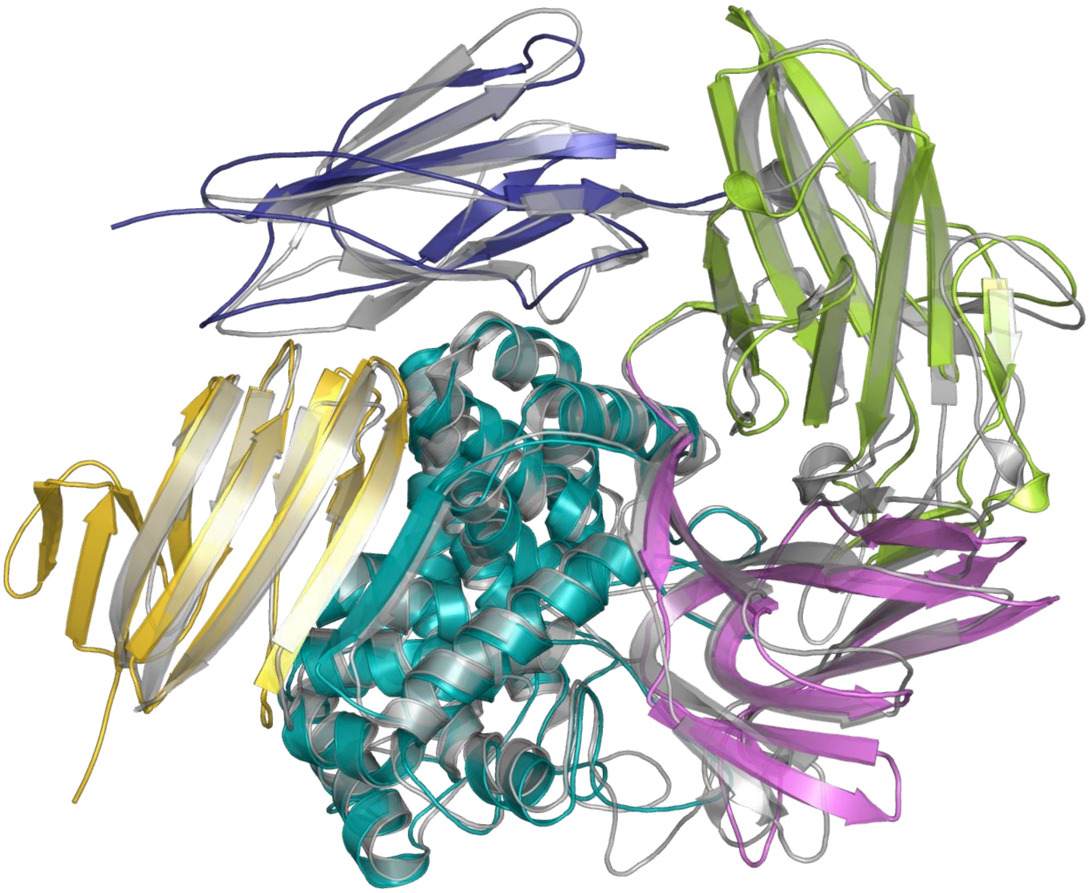

Figure S2. The ArRha 3D model has been superimposed onto homologous enzyme AtRha rhamnosidase from *A. terreus* (PDB: 6gsz), rendered in grey to indicate the overlay.

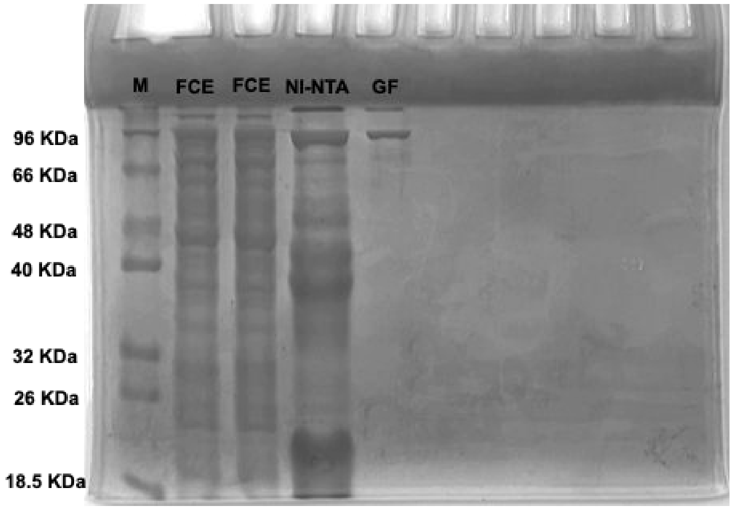

Figure S3. SDS-PAGE analysis of ArRha expressed in *E. coli* BL21 (DE3) LEMO. Lane M: molecular weight marker, lanes FCE: Free Cell Extract, lane Ni-NTA: ArRha purified by nickel-affinity chromatography, Lane GF: purified protein by Gel Filtration.

Table S1. Purification table from 2-liters growth.

Sample	Vol (mL)	Concentration (mg/mL)	Total protein (mg)	Specific activity (U/mg)	Total units	Purification fold	Yield %
***FCE**	90.00	18.57	1671.30	0.16	267.41	1.00	100.00
**His-trap**	6.00	1.86	11.16	2.50	27.90	15.63	10.43
***GF**	10.00	~ 0.20	2.00	~ 12.00	25.70	80.31	9.61

*FCE: Free cell extract, GF: Gel filtration. Assays were performed at 75°C, pH 6.5, see Materials and Methods section.

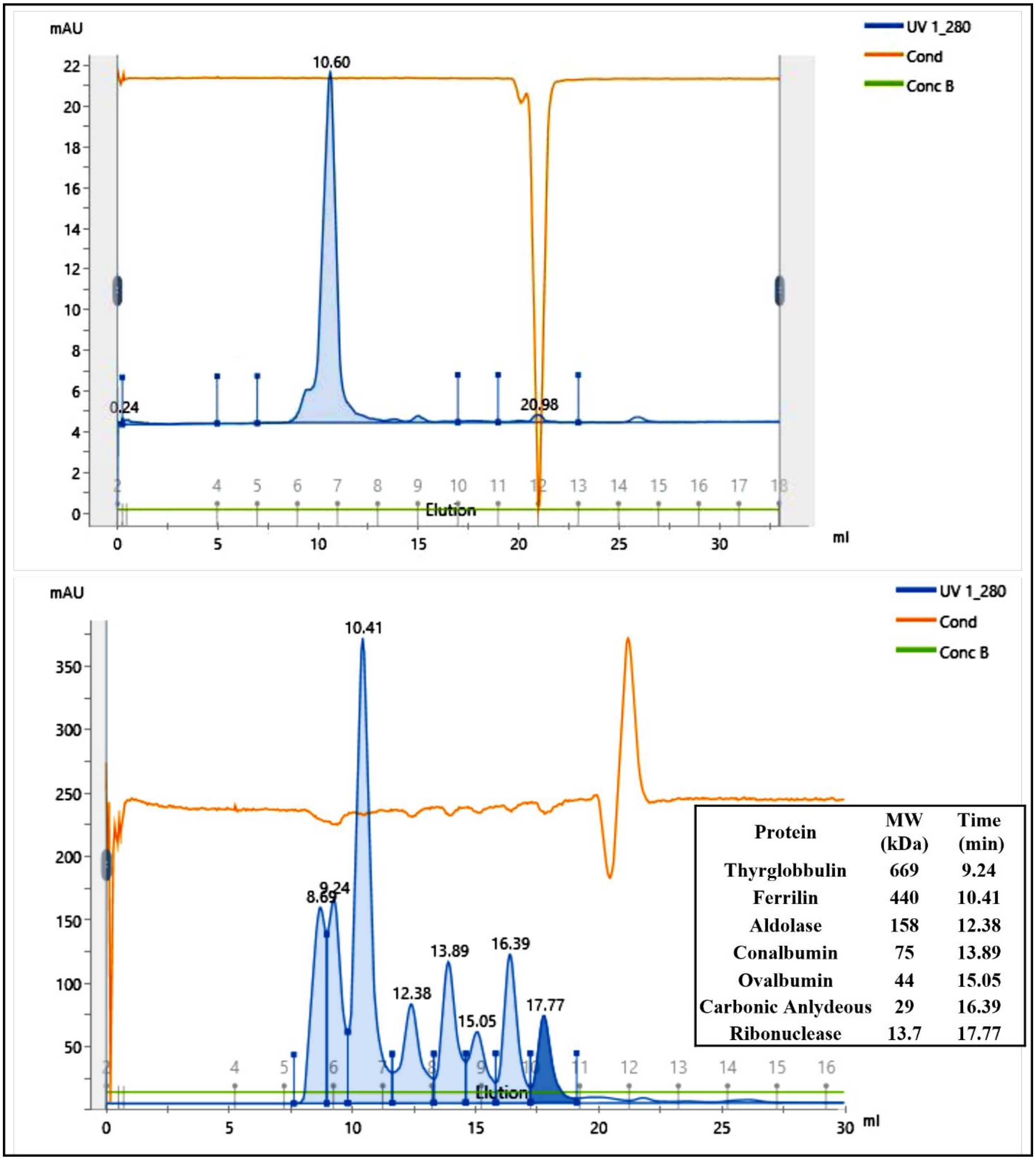

Protein	MW (kDa)	Time (min)
Thyrglobbulin	669	9.24
Ferrilin	440	10.41
Aldolase	158	12.38
Conalbumin	75	13.89
Ovalbumin	44	15.05
Carbonic Anlydeous	29	16.39
Ribonuclease	13.7	17.77

Figure S4. FPLC chromatogram displaying the elution peak of recombinant ArRha and the profiles for molecular weight standards. The native molecular mass was determined using a $\log_{10}$ molecular weight versus elution volume calibration curve generated from standard proteins ArRha.

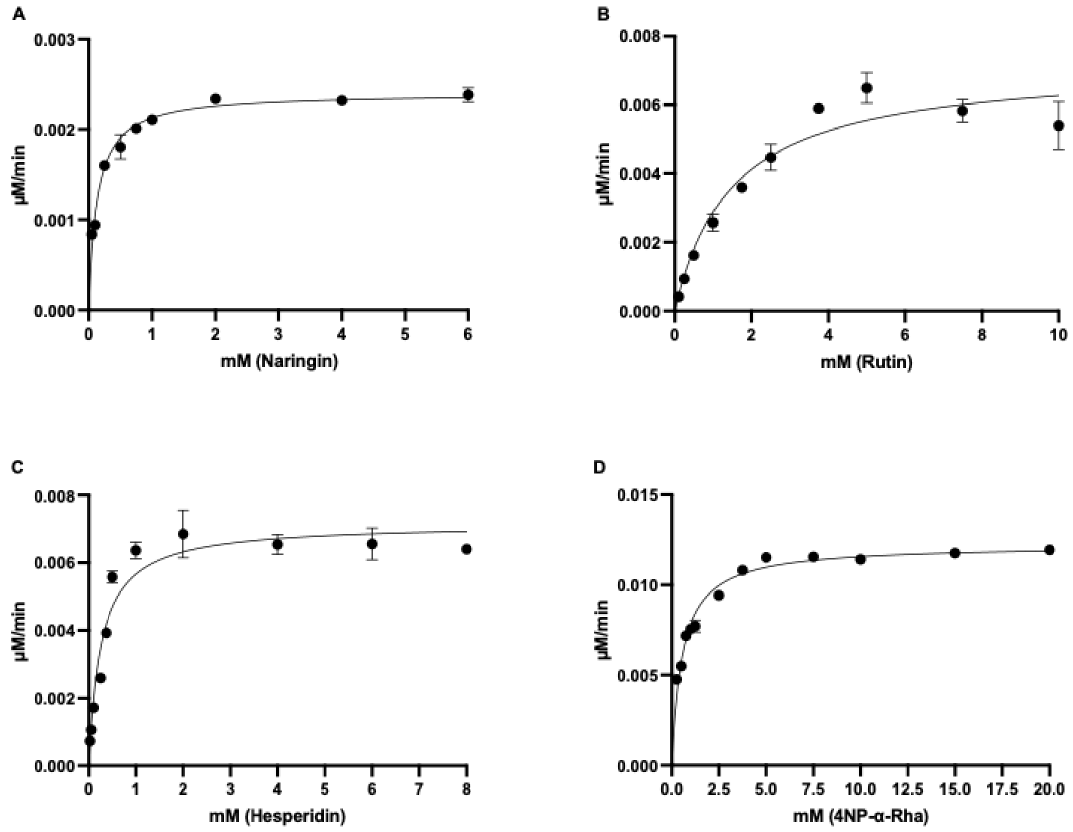

Figure S5. Michaelis-Menten kinetic curves for (A) naringin, (B) rutin, (C) hesperidin and (D) 4NP-α-Rha.

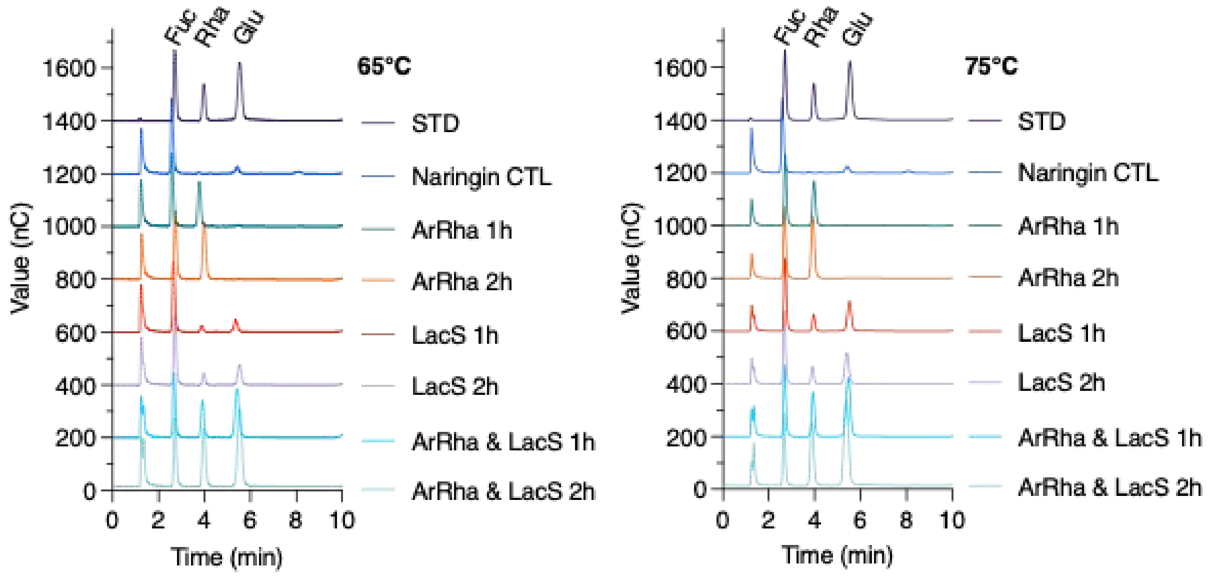

Figure S6. HPAEC-PAD chromatographic profiles showing the identification of rhamnose and glucose released following the enzymatic biotransformation of naringin for 1 and 2 hours at 65 and 75°C.
